# Empowering macrophages: the cancer fighters within the tumour microenvironment in mantle cell lymphoma

**DOI:** 10.3389/fimmu.2024.1373269

**Published:** 2024-03-19

**Authors:** Patrick Nylund, Anna Nikkarinen, Sara Ek, Ingrid Glimelius

**Affiliations:** ^1^ Department of Immunology, Genetics and Pathology, Cancer Precision Medicine Unit, Uppsala University, Uppsala, Sweden; ^2^ Department of Immunotechnology, Lund University, Lund, Sweden; ^3^ Division of Clinical Epidemiology, Department of Medicine, Karolinska Institute, Stockholm, Sweden

**Keywords:** mantle cell lymphoma, macrophages, tumor microenvironment, immunotherapy, car-t, CAR-M, drug resistance

## Abstract

In Mantle Cell Lymphoma (MCL), the role of macrophages within the tumour microenvironment (TME) has recently gained attention due to their impact on prognosis and response to therapy. Despite their low absolute number in MCL tumour tissue, recent findings reveal an association between the levels of macrophages and prognosis, consistent with trends observed in other lymphoma subtypes. M2-like macrophages, identified by markers such as CD163, contribute to angiogenesis and suppression of the immune response. Clinical trials with MCL patients treated with chemoimmunotherapy and targeted treatments underscore the adverse impact of high levels of M2-like macrophages. Immunomodulatory drugs like lenalidomide reduce the levels of MCL-associated CD163^+^ macrophages and enhance macrophage phagocytic activity. Similarly, clinical approaches targeting the CD47 “don’t eat me” signalling, in combination with the anti-CD20-antibody rituximab, demonstrate increased macrophage activity and phagocytosis of MCL tumour cells. Cell-based therapies such as chimeric antigen receptor (CAR) T-cell have shown promise but various challenges persist, leading to a potential interest in CAR-macrophages (CAR-M). When macrophages are recruited to the TME, they offer advantages including phagocytic function and responsiveness to microenvironment alterations, suggesting their potential as a manipulable and inducible alternative when CAR T-cell therapies fails in the complex landscape of MCL treatment.

## Introduction

Mantle cell lymphoma (MCL) corresponds to about 5% of all non-Hodgkin’s lymphomas (NHL) and originates from the malignant transformation of B-cells within the mantle zones of the lymph nodes. MCL can also arise from antigen-experienced B-cells that have undergone immunoglobulin heavy-chain variable (IGVH) somatic hypermutations elsewhere in the lymphatic tissue (1). Due to disease heterogeneity, MCL is challenging to treat and remains mostly incurable, with patients exhibiting a median overall survival of 1.8 to 9.4 years depending on their individual genetic and pathological risk factors (2–4). Furthermore, patients often relapse as a result of drug resistance, leading to an overall poor prognosis (5, 6). Interestingly, recently developed immunotherapies including anti-CD19 chimeric antigen receptor T (CAR-T) cell therapy (7) and bispecific antibodies (8) have shown promising treatment outcomes at relapse. Thus, in the present review we aim to elucidate the current knowledge of the MCL tumour microenvironment (TME) and discuss how targeting these systems could improve patient response and survival even further.

### Established prognostic factors in mantle cell lymphoma

The primary oncogenic event that takes place in MCL cells is t(11;14)(q13;q32), but this genetic aberration alone is not enough to drive lymphoma pathogenesis (9, 10). Instead, MCL pathogenesis is underpinned by increased genomic instability by mutations in genes that regulate cell cycle progression, such as ATM, TP53 and RB1, as well as genes that regulate DNA repair, NF-κB signalling and apoptosis. As such, detection of TP53 mutations in the tumour cells is one of the strongest negative prognostic markers in patients. In the absence of these specific gene mutations, transcriptional dysregulation can also cause aberrant protein function, as exemplified by the poor prognosis for patients with MYC overexpression (11, 12).

Markers for poor prognosis that are used in the clinic include a high mantle cell lymphoma international prognostic index (MIPI), high proliferation rate measured with Ki-67, blastoid and/or pleomorphic histology, as well as an abnormal karyotype (13–17). However, it has become clear that patient outcome cannot be determined by studying only the disease-specific cell types, but must include the much wider system of supporting and interacting cells that constitute the TME (18–20). The tumour architecture, presence and composition of inflammatory cells in the tumour tissue, and soluble biomarkers are important aspects of the TME. However, markers reflecting the TME are not yet being used as prognostic factors in MCL care. Despite this, the TME is highly involved in the established treatments, and the prognostic factors of the TME are becoming increasingly important to consider with the future expansion of immunotherapy in MCL treatment.

### Current therapeutic approaches in mantle cell lymphoma

In younger MCL patients, the standard therapy has consisted of monoclonal anti-CD20 antibody rituximab together with intensive chemotherapy, i.e., cyclophosphamide, doxorubicin, vincristine prednisone (CHOP) and cytarabine or oxaliplatin, followed by an autologous stem cell transplantation (21). However, the introduction of novel targeted drugs, i.e., the Bruton tyrosine kinase inhibitor (BTKi) ibrutinib as maintenance after induction chemotherapy, has proven to be as effective with our without autologous stem cell transplantation (22). On the other hand, for elderly patients there is no general agreement on treatment internationally (23), but most patients have received chemotherapy (CHOP or bendamustine) in combination with rituximab.

A new era utilizing targeted drugs and potentially omitting chemotherapy is emerging in MCL care (24–26). The covalent BTKi:s ibrutinib, acalabrutinib and zanubrutinib have shown efficacy in relapsed patients (27). However, the survival benefit when using BTKi as a first-line therapy compared to chemotherapy is unknown, and we are eagerly awaiting the results for the randomized ENRICH trial comparing chemotherapy to first-line BTKi in elderly patients. A key question is whether BTKi can replace chemotherapy or add to its efficacy. Furthermore, the non-covalent BTKis pirtobrutinib and nemtabrutinib have also proven effective when used in conjunction with covalent BTKis at disease progression for various B-cell malignancies (28, 29).

The novel field of CAR-T cell therapy and bispecific antibodies is the next step in treatment. The CAR-T cell-based therapies tisagenlecleucel (Kymriah), axicabtagene ciloleucel (Yescarta), brexucabtagene autoleucel (Tecartus) and lisocabtagene maraleucel (Breyanzi) have been FDA approved for treatment of various hematological malignancies. In addition, the bispecific antibody-based therapy epcoritamab has also recently been FDA approved, but is not yet readily available in many countries due to reimbursement regulations. Immunotherapy has revolutionized the care of MCL patients relapsing after chemoimmunotherapy and BTKi (7, 30, 31). Summarized in **Tables 1**, **2** are a selection of ongoing and actively-recruiting clinical trials on the next generation of CAR-T cell therapy in MCL (**Table 1**) as well as a table of ongoing trials on different bispecific antibodies in MCL (**Table 2**). Resistance mechanisms of BTKi have been attributed to mutations in the BTK binding site as well as to various somatic mutations. However, for novel drugs such as the new generation of BTKi, CAR-T cell therapy and bispecific antibodies, specific resistance mechanisms are largely underexplored. The most described cause is loss of the target molecule, usually CD19, but other resistance mechanisms can include exhaustion of the CAR-T cells. It now remains to be determined which local factors in the TME and more general factors of the individual’s immune response and immune state can influence the response to treatment (**Table 3**).

**Table 1:** Selected planned, ongoing actively recruiting CAR-T cell clinical trials in MCL on clinicaltrials.gov February 2024.

NCT/EUCT ID	Targeted disease	Clinical Trial	Therapy
NCT04484012	Relapse & Refractory MCL	Phase II	CD19 CAR-T & acalabrutinib
NCT05934838	B-cell Lymphomas	Phase I	CAR-T and tazemetostat
NCT05020392	B-cell Lymphomas	Phase III	Anti-CD19 CAR-Engineered T Cells with BTKi
NCT06026319	Relapsed/Refractory Non-Hodgkin Lymphomas	Phase I	CD79b-19 CAR T Cells
NCT05495464	High Risk MCL	Phase I	Acalabrutinib plus rituximab followed by brexucabtagene autoleucel
NCT04007029	Relapse & Refractory Lymphomas	Phase I	CAR-20/19-T Cells
NCT05444322	Relapse & Refractory Lymphomas	Phase I	CAR-T cell RD14-01
NCT05370430	Relapse & Refractory Non-Hodgkin Lymphomas	Phase I	BAFFR-Targeting CAR-T Cells
NCT03676504	CD19+ Lymphoid Disease	Phase I/II	Third generation CD19 CAR-T
2022-502405-15-00	CAR-T-cell treatment after an abbreviated induction therapy with rituximab and ibrutinib	Phase II	anti-CD19 CAR-T
NCT06002659	B Cell Lymphoma	Phase I/II	anti-CD20(NAP)
NCT05588440	Relapsed or Refractory B-Cell Malignancies	Phase I/II	ROR1 targeting autologous CAR T

**Table 2:** Selected planned, ongoing and actively recruiting bispecific antibody clinical trials in MCL on clinicaltrials.gov February 2024.

NCT/EUCT ID	Targeted disease	Clinical Trial	Therapy
NCT06054776	MCL	Phase II	Glofitamab (anti-CD3/CD20) with acalabrutinib
NCT04703686	Relapse/​Refractory Lymphomas after CAR T-cells Therapy	Phase II	Glofitamab
NCT00014560	Refractory or Relapsed Non-Hodgkin’s Lymphoma	Phase I	4G7XH22 (anti-CD19/CD30)
NCT05861050	Newly Diagnosed High Risk Mantle Cell Lymphoma	Phase I/II	Glofitamab with obinutuzumab, venetoclax, and lenalidomide
2023-503206-37-00	Relapse/Refractory MCL post BTKi therapy	Phase III	Glofitamab vs rituximab + bendamustine or rituximab + lenalidomide
NCT04763083	ROR1+ Malignancies	Phase I	NVG-111 (ROR1/CD3)
NCT03625037	Refractory B-Cell Lymphoma		Epcoritamab (anti-CD20/CD3)
NCT06192888	MCL	Phase I	Glofitamab with lenalomide
NCT05833763	MCL with prior BTKi treatment	Phase II	Glofitamab and pirtobrutinib
NCT06084936	Refractory or Relapsed MCL	Phase III	Glofitamab
NCT03888105	B-cell Non-Hodgkin Lymphoma	Phase II	Odronextamab

**Table 3:** Example of drug resistance mechanisms in mantle cell lymphoma.

Therapy	Target Molecule	Mechanism of resistance	Reference
Rituximab	Anti-CD20	CD20 degradation due to cell adhesion	(32)
Venetoclax	BCL2	CD40 expression promote BCL expression inhibiting pro-apoptotic proteins	(33–35)
CAR-T Therapy	Anti-CD19	Loss of target marker or insufficient CAR-T cell expansion. Exhausted CAR-T cells, surrounding immunosuppressive TME	(7, 36)
BTKi	Bruton tyrosine kinase	Mutations in BTK binding site as well as mutations in CCND1, TRAF2-3, MAP3K14, CARD11 and MYD88	(30, 31, 37–41)

## The tumour microenvironment causing resistance to therapy

**Figure 1:**
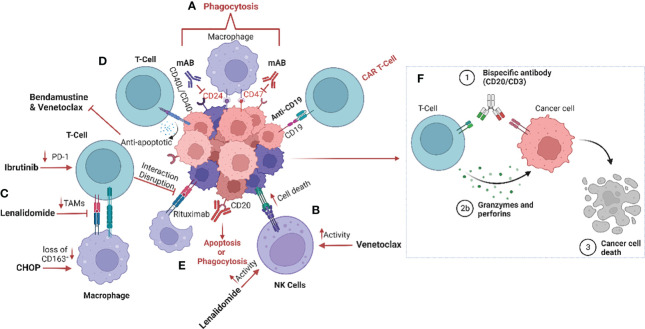
Schematic representation of the impact of treatment regimens on the MCL tumour microenvironment (TME). **(A)** The current strategies targeting the tumour microenvironment (TME), especially macrophages, include anti-CD24 and anti-CD47 monoclonal antibodies to enhance phagocytosis by inhibiting the “don’t eat me’ signalling (42). **(B)** Furthermore, treatment with lenalidomide and venetoclax increase natural killer (NK) cell activity and **(C)** reduce tumour-associated macrophages (TAMs) within the TME (35, 43–45). **(D)** CD40/CD40L interaction induces venetoclax and bendamustine resistance by promoting release of anti-apoptotic signalling such as BCL family proteins (33–35). **(E)** CD20 monoclonal antibody therapy results in the induction of phagocytosis, and more recently **(F)** bispecific antibodies promote the interaction between T-cells and tumour cells, causing T-cell induced cytotoxicity (46). Image was created with biorender.com.

Drug resistance has previously been attributed to cellular processes within the TME. MCL cells are dependent on the TME for increased proliferative capacity, cellular survival and immune system evasion. As such, there is a rationale for targeting the TME in combination with conventional and novel immunotherapeutic drugs to improve patient response and outcome (**Figure 1**). Recent studies have shown that MCL cells invading the bone marrow became resistant to rituximab as a result of losing CD20 expression, while the tumour cells in the lymph node tissue maintained their extensive CD20 expression (32). Here, the process of CD20 degradation was attributed to bone marrow stromal cell adhesion (32). Combinatorial inhibition of focal adhesion kinase and ibrutinib has been shown to overcome resistance to ibrutinib in MCL cells (47) (**Table 3**).

It has previously been established that MCL cells are dependent on the CD40-CD40L interaction between the tumour cells and T-cells, and that this interaction plays an important role in promoting MCL cell survival (**Figure 1**). MCL cells that express CD40 induce the expression of the B-cell lymphoma (BCL) genes, a family of anti-apoptotic proteins which inhibit mitochondrial apoptotic priming and counteract pro-apoptotic proteins (33, 34) (**Table 3**), leading to resistance to the BCL2 inhibitor venetoclax (35) (**Figure 1**). However, treatment with the anti-CD20 antibody obinutuzumab can overcome venetoclax resistance by counteracting NF-κB-induced Bcl-XL expression (33, 35) (**Figure 1**). Interestingly, obinutuzumab treatment of MCL cells impaired p52 expression and reduced the expression of NF-κB target genes in cells with high expression of CD20 (33). Venetoclax also reduces the number of T regulatory cells (Tregs), lowers PD-1 expression in T-cells and increases natural killer (NK) cell function (**Figure 1**). Many MCL cells overexpress the transcription factor SOX11 which promotes expression of another co-stimulatory molecule, such as CD70 (48). CD70 bind to CD27 on T-cells in non-Hodgins lymphoma, resulting in T-cell exhaustion (49).

Different T-cell-engaging treatments such as CAR-T cells and bispecific antibodies function by targeting the TME directly or indirectly. In CAR-T cell treatment, T-cells are harvested from the patient, modified, and returned to take part in targeting the lymphoma cells and inducing anti-tumoral immune responses. Anti-CD19 CAR-T cell therapy has been approved for treatment of MCL patients that relapse after BTK treatment, who typically have a poor prognosis (7) (**Figure 1**). However, CAR-T cell treatment failures are frequent due to loss of target marker or insufficient CAR T-cell expansion (**Table 3**). Different armed CAR-T cells, the addition of co-stimulatory factors, production of CAR-T cells from T-cells harvested earlier in the disease course and dual binding sites are ways to improve the efficacy of CAR-T cells (36). Another way of improving efficacy is to use combinatorial therapies. Bispecific antibody therapy after CAR-T treatment is also currently undergoing evaluation of efficacy when loss of target for the CAR-T cells occurs and in abrogating T-cell exhaustion. The bispecific antibodies (anti-CD20/CD3) bind T-cells to lymphoma cells, facilitating T-cell engagement and activation of antibody-dependent cellular cytotoxicity (ADCC) against the lymphoma cells (50) (**Figure 1**). However, resistance is also observed in treatments with bispecific antibodies, particularly over time.

## Macrophages in MCL therapy

### Macrophages in MCL

Macrophages are a crucial component of the immune infiltrate in MCL. These immune cells are often referred to as tumour-associated macrophages (TAMs) when found within the TME. TAMs are highly plastic and can exhibit distinct functional states, with the two primary polarization states being M1-like (pro-inflammatory and anti-tumoral) and M2-like (anti-inflammatory and pro-tumoral). This division is likely an over-simplification but still useful. CD32, CD64, CD68, CD80 and CD86 have all been reported as surface markers for M1-like macrophages (51). M2-like macrophages are most commonly assessed using CD163, a scavenger receptor on the macrophage surface that can be released into the surrounding tissue or bloodstream upon activation (52). Another cell surface marker for identifying M2-like macrophages that are induced as a result of inflammation is CD206 (53). In addition, M1 and M2 cellular differentiation is highly dependent on several TME conditions such as hypoxia and hypoglycaemia. TAM utilize lipid metabolic pathways to sustain energy needs which can promote or inhibit tumour pathogenesis (54). Furthermore, other studies suggests that TAMs are subjected to the Warburg effect by utilizing oxidative phosphorylation rather than aerobic glycolysis for ATP production, which promotes the differentiation of M2-like macrophages. This provides a rationale to target the lipid metabolic pathway and/or mechanism of the Warburg effect to promote M1-like phenotypic differentiation of TAM which could promote response to conventional immunotherapies and reduce resistance (55, 56).

Although absolute numbers of macrophages are low in MCL tumour tissue, their presence was recently shown to be associated with poorer prognosis (19). This is not surprising, since it is known from the literature that macrophages correlate with poorer prognosis in many lymphoma subtypes (46, 57–60). However, this knowledge has only recently begun being utilized in predicting prognosis (19), identifying soluble markers (18) and enhancing the efficacy of novel immunotherapy treatments in the MCL setting (61).

### Targeting macrophages in MCL

Macrophages play an important role in regulating the response to classical chemotherapy (62). The underlying biological mechanisms of CHOP response, which has been the backbone in MCL treatment, are also dependant on alteration of the microenvironment. In mice with diffuse large B-cell lymphoma (DLBCL) that were treated with CHOP and R-CHOP (addition of rituximab to standard CHOP therapy), the proportion of CD163^+^ macrophages were lower after both treatments, indicating a repolarization of M2-like macrophages to M1-like with these regimens (**Figure 1**) (63). It has however not yet been investigated how much of this effect was due to the glucocorticoid prednisolone, which itself has a modulatory effect on macrophages. In a wound repair model, macrophages treated with glucocorticoids upregulated CD163 and CD206 expression, thus demonstrating polarization towards anti-inflammatory M2-like macrophages which also showed a decreased migration ability (64). Although glucocorticoids are widely used in oncology, the effects on the TME are not yet fully understood and the elucidation of their impact will require further investigation.

In MCL, M2-polarization of macrophages is common, contributing to angiogenesis and inhibition of the immune response (65). Co-culture of monocytes with MCL cells isolated from newly diagnosed patient biopsies promotes M2-like macrophage differentiation with increased expression of CD163, which is associated with poor patient outcome and blastoid morphology, independent of T-cell infiltration (19). Clinical trials of NHL (including MCL patients treated with rituximab, intensive chemotherapy and autologous stem cell transplantation) showed a poorer outcome for patients exhibiting a high presence of M2-like macrophages, and it was suggested that infiltration of these macrophages could have an impact on the response to anti-CD20 therapy (19, 57, 60, 66). Furthermore, MCL cells secrete colony stimulating factor 1(CSF1), which promotes the differentiation of monocytes into M2-like CD163^+^ macrophages and thereby stimulates MCL proliferation and survival (67). Depletion of macrophages by blocking the CSF1/CSF1R axis *in vivo* has been demonstrated to reduce differentiation and survival of M2-type macrophages (68). Other drugs such as the BTKi ibrutinib also impacts the TME by exerting an immunomodulatory effect through regulation of tumour-infiltrating macrophages (69). In addition, ibrutinib downregulates PD-1 expression on T-cells, disrupts communication between MCL cells and macrophages, and impairs macrophage phagocytic function (70) (**Figure 1**).

We have previously confirmed the prognostic value of CD163 by measuring soluble CD163 (sCD163) in the serum of patients with MCL. High sCD163 was associated with shorter progression-free survival and poor outcome in patients treated with rituximab, ibrutinib and lenalidomide in a phase 2 clinical trial (18). Although sCD163 levels were higher in patients with p53-deficient tumours, the prognostic value of sCD163 was independent of TP53 mutations and other clinical factors such as MIPI, Ki-67 or blastoid morphology, while low sCD163 levels could identify patients with a favourable prognosis (18). Various clinical approaches have been investigated to target M2-like macrophages and disrupt MCL proliferative advantage. One such approach is lenalidomide, which is an immunomodulatory drug targeting cereblon (CRBN), a highly conserved gene that promotes ubiquitination. Lenalidomide is currently approved for treatment of multiple myeloma (MM) and MCL (71), and has been found to affect many cells of the TME, including macrophages. Accordingly, lenalidomide reduced the abundance of TAMs (43), enhanced macrophage phagocytic activity and increased macrophage communication with CD8^+^ T-cells (44) (**Figure 1**). In addition, treatment with lenalidomide has a clinical benefit in relapsed MCL patients, defined by increased NK cell activity (45). Just recently, the combination of lenalidomide, venetoclax and rituximab was shown to be feasible and efficacious in relapsed MCL patients, using a minimal residual disease (MRD)-driven treatment design (72).

To be able to study the MCL lymph node signalling, heterogeneity and TME, 3D models of MCL-spheroids have been created (70). These spheroids caused monocytes to differentiate into M2-like macrophages, which were interestingly reprogrammed into a more immunogenic phenotype upon blockade of the chemokine receptor CCR1 (53, 70). Prior studies have shown that co-culturing MCL cells with TAMs assisted the tumour cells to avoid phagocytosis. However, dual inhibition of CD24 and CD47, two molecules enhancing the “don’t eat me” signal, in combination with rituximab induced increased macrophage activity and promoted phagocytosis in MCL cells (42) (**Figure 1**). Interestingly, other “don’t eat me” signalling pathways have been identified and include the PD-1/PD-L1, MHC-I/LILLRB1/2 and CD24/SIGLEC-10 axes. Targeting these mechanisms have been shown to induce activation of macrophages and increase phagocytosis (73–75). In addition, monoclonal antibodies targeting CD47 on the tumour cells have been demonstrated to activate macrophages and induce phagocytosis of the tumour cells. Furthermore, ongoing clinical trials in MCL are exploring the targeting of “don’t eat me” signalling by utilizing anti-CD24 therapy. CD24-expressing cells promotes immune response escape by reprogramming macrophages not to target them for phagocytosis (NCT05888701). Clinical studies have demonstrated the importance of blocking the “don’t eat me” signalling pathways in various haematological malignancies including MCL by specifically targeting the CD47/SIRPα axis (76). Thus, utilizing CD47-targeting bispecific antibodies to enhance the response may be a promising therapeutic intervention to increase treatment efficiency. Preclinical evaluation of CD47/PD-1, CD47/CD33 CD47/PD-L1, CD47/CD20, CD47/CD19 and CD47/CD20 are currently ongoing (77–82).

### CAR-macrophage therapy

Despite the relatively high success rate of CAR T-cell therapies in haematological malignancies, numerous challenges remain, including a lack of specific targets. Furthermore, CAR T-cell trafficking and infiltration to tumour sites is difficult due to the abnormal vasculature and dysregulated adhesion matrices (83). Another limitation in CAR T-cell therapies is that the TME includes Tregs, TAMs, tumour-associated fibroblasts and myeloid-derived suppressor cells that create a hostile environment by secreting immunosuppressive cytokines that impair CAR T-cell function. Antigen escape and lack of CAR T-cell expansion and persistence during treatment can also limit the effectiveness of the therapy (84). Direct TAM-targeted therapies have been reported but are highly dependent on the presence of either activating or suppressive macrophage markers, and can cause adverse effects. Clinical data of these therapies is currently lacking for MCL.

**Figure 2:**
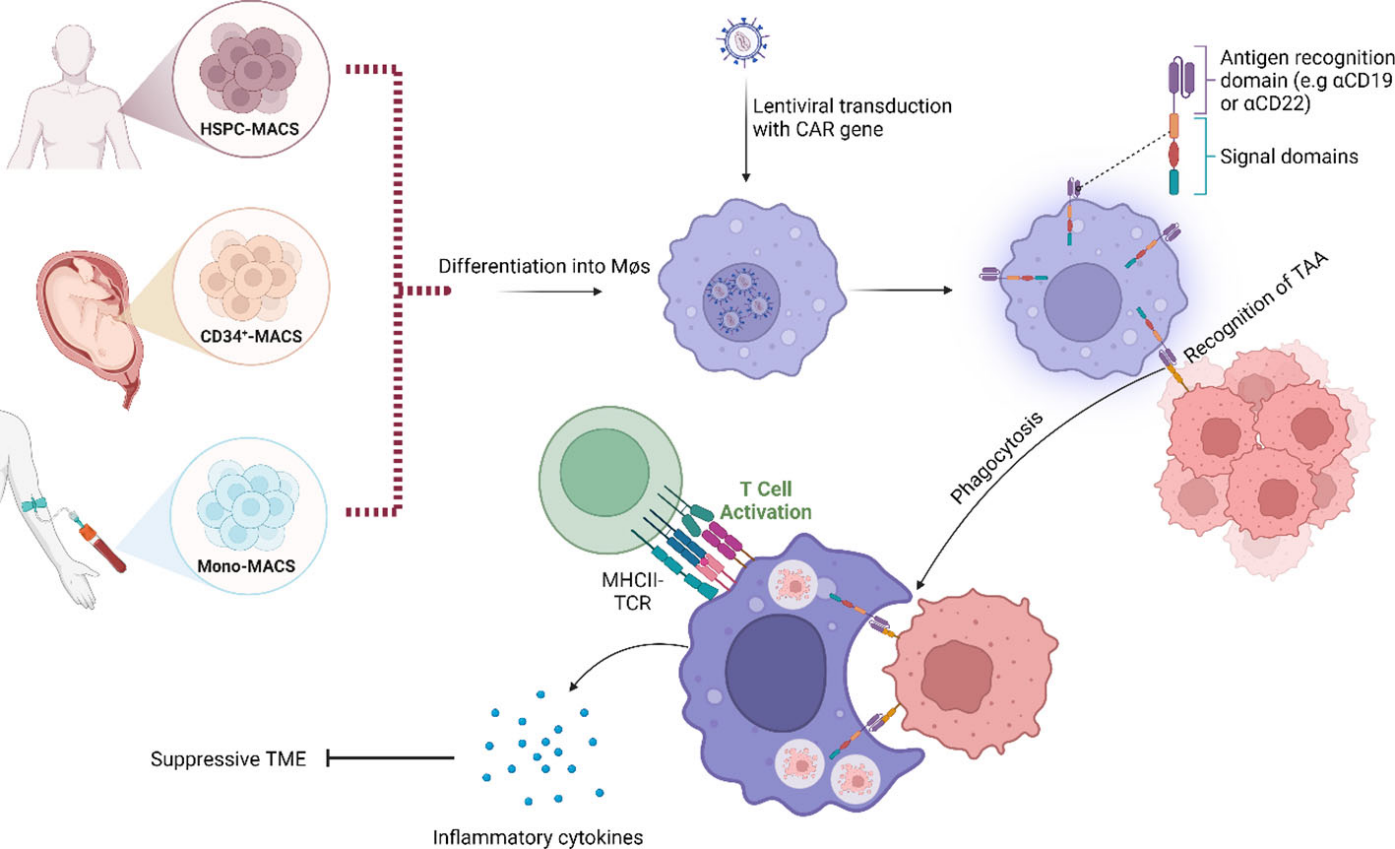
Schematic representation of the modes of action of chimeric antigen receptor–macrophages (CAR-M). Instead of using a T-cell as carrier for the CAR construct, a macrophage is used. Binding of tumour-associated antigen by CAR-M induces phagocytosis, accompanied by the release of inflammatory cytokines that activate additional immune responses within the tumour microenvironment (TME). Examples of downstream effects include T-cell-mediated anti-tumour immune responses. Image was created with biorender.com.

Recently, the development of CAR-macrophages (CAR-M) has become increasingly interesting as a potential solution to the challenges observed with TAM-targeting and CAR T-cell therapies. As of now, CAR-M is not yet in clinical trials for MCL, but since macrophages are recruited to the TME as a result of the production of cancer-associated cytokines and hypoxic conditions, it has been suggested that CAR-M could be utilized to overcome the problem of poor capacity for trafficking and infiltration (85). Early precursors of CAR-M were the simultaneous blocking of CD47/SIRPα which have been shown to promote phagocytotic activity with high specificity against the tumour antigen (86). In fact, next generation CAR-M therapies targeting CD19 promoted phagocytosis in an antigen-specific manner and converted M2-like macrophages to M1-like. Interestingly, these CAR-M also secreted proinflammatory cytokines and chemokines, upregulated antigen presentation, promoted activation of cytotoxic T-cells and overcame the immunosuppressive microenvironment *in vivo* (87, 88). Additional preclinical studies of CAR-M include the target antigens CD5, CD22, HER2, CCR7 and ALK (89, 90). Another potential advantage of this therapy is that, even when in the immunosuppressive M2-like state, macrophages still maintain the function of phagocytosis. It has also been suggested that macrophages are more responsive to alterations in the TME, making them easier to manipulate and induce than CAR T-cells (91) (**Figure 2**). However, several challenges remain on this front, including finding an appropriate source of macrophages for modification, since immortalized cells cannot be applicable for clinical utility while blood and bone marrow samples cannot be modified efficiently (**Figure 2**). Interestingly, induced pluripotent stem cells (iPSCs)-derived macrophages have been demonstrated to recapitulate similar anti-tumour function described by *Klichinsky et al.* (87), in both solid and haematological malignancies (92). Currently, there are two ongoing phase I clinical trials evaluating the potential of CAR-M in HER2-overexpressing solid tumours and advanced gastric cancer with peritoneal metastases (NCT04660929 & NCT06224738), but no clinical trials have been performed in MCL to date. To conclude, the strengths of CAR-M include increased tumour infiltration, reduction of TAMs and alteration of TAM phenotypes within the TME. CAR-M can also stimulate increased phagocytosis and promote antigen presentation, resulting in improved cytotoxic T-cell-mediated anti-tumour effects with limited off-target effects. As such, the potential of CAR-M should be further explored.

## Discussion

Herein, we comprehensively explore the intricate relationship between the TME and its profound impact on treatment response, resistance mechanisms, and ultimately patient outcomes, with a particular focus on macrophages. We highlight the significance of macrophages in treatment dynamics, and their dual role in either promoting or inhibiting therapeutic responses. TAMs are highly plastic and have the ability to adopt distinct functional states, notably M1-like with pro-inflammatory and anti-tumoral characteristics, or M2-like with anti-inflammatory and pro-tumoral features. This polarization dynamic could be essential as a determinant in treatment outcomes.

We have summarised the current treatment strategies and stipulated the many challenges associated with choice of drug. The next objective in immunotherapy is to investigate if, and how, a patient’s individual immune state would impact the degree of side effects caused by different immunotherapies. We are constantly learning more about effective side effect management, but patients are often still hospitalized for safety reasons. For example, high-dose steroids are required for prophylaxis, and patients can suffer from severe complications such as cytokine release syndrome and immune effector cell-associated neurotoxicity syndrome. While the exact mechanisms of these immune-mediated toxicities are not clearly understood, emerging pre-clinical and clinical studies have revealed the pivotal role of myeloid cells, particularly macrophages, as contributors to the efficacy of treatments but also as crucial mediators of toxicity (93).

Immunotherapy takes advantage of the TME to defeat the malignant cells, as opposed to focusing on the disruption of such interactions. CAR-T cell therapies and bispecific antibodies have already paved the way in relapsed/refractory disease in various haematological malignancies and the next step is testing them as first-line treatment combinations for high-risk patients. M2-like macrophages are involved in CAR-T cell therapy response both *in vivo* and *in vitro*, leading to CAR-T cell therapy failure and disease progression in DLBCL (94), likely having similar activities in MCL. Targeting macrophages with bispecific antibodies is a therapeutic possibility, but has currently not been evaluated in a clinical setting (95). Furthermore, reprogramming of pro-tumoral macrophages to anti-tumoral macrophages, as well as utilization of CAR-M are potential novel approaches in MCL therapy (95). The balance between achieving efficacy and the potential toxicity of macrophage-targeted therapies will however be challenging, since they exhibit a high degree of diversity and plasticity and can adopt different functional states in response to the TME. Modulating macrophage activity may trigger inflammatory responses, leading to cytokine release syndrome or other immune-related adverse events, and as such balancing therapeutic efficacy with the risk of systemic inflammation is crucial. Similarly, macrophages can play both pro-tumor and anti-tumor roles, which in combination with a lack of target specificity can lead to unintended effects on other tissues or immune cells.

In summary, the TME plays a large role in determining the response to both conventional but also next generation therapies for MCL, where a high presence of CD163^+^ macrophages have a negative prognostic impact. It is evident that by combining the therapies now in clinical utility with strategies directly targeting the TME, patient outcome could be improved, but additional clinical evaluation is required.

## Author contributions

PN: Conceptualization, Project administration, Visualization, Writing – original draft, Writing – review & editing. AN: Project administration, Writing – original draft, Writing – review & editing. SE: Writing – original draft, Writing – review & editing. IG: Conceptualization, Funding acquisition, Project administration, Writing – original draft, Writing – review & editing.
